# Model of Anisotropic Reverse Cardiac Growth in Mechanical Dyssynchrony

**DOI:** 10.1038/s41598-019-48670-8

**Published:** 2019-09-03

**Authors:** Jayavel Arumugam, Joy Mojumder, Ghassan Kassab, Lik Chuan Lee

**Affiliations:** 1 Department of Mechanical Engineering, Michigan State University, East Lansing, USA; 2 California Medical Innovations Institute, San Diego, CA USA

**Keywords:** Cardiac hypertrophy, Biomedical engineering

## Abstract

Based on recent single-cell experiments showing that longitudinal myocyte stretch produces both parallel and serial addition of sarcomeres, we developed an anisotropic growth constitutive model with elastic myofiber stretch as the growth stimuli to simulate long-term changes in biventricular geometry associated with alterations in cardiac electromechanics. The constitutive model is developed based on the volumetric growth framework. In the model, local growth evolutions of the myocyte’s longitudinal and transverse directions are driven by the deviations of maximum elastic myofiber stretch over a cardiac cycle from its corresponding local homeostatic set point, but with different sensitivities. Local homeostatic set point is determined from a simulation with normal activation pattern. The growth constitutive model is coupled to an electromechanics model and calibrated based on both global and local ventricular geometrical changes associated with chronic left ventricular free wall pacing found in previous animal experiments. We show that the coupled electromechanics-growth model can quantitatively reproduce the following: (1) Thinning and thickening of the ventricular wall respectively at early and late activated regions and (2) Global left ventricular dilation as measured in experiments. These findings reinforce the role of elastic myofiber stretch as a growth stimulant at both cellular level and tissue-level.

## Introduction

Mechanical dyssynchrony of the heart such as left bundle branch block (LBBB) is caused by the asynchronous electrical activity of the left ventricle (LV) through slow conduction across the myocytes instead of through the fast conduction Purkinje system. Besides acute changes like QRS prolongation, reduction in wall motion and changes in blood flow^1^, abnormal contraction of the dyssynchronous heart can lead to long-term ventricular remodeling if left untreated. Patients with LBBB not only have an increased risk of developing cardiac disease^2^, such as hypertension, congestive heart failure, but also have a higher mortality rate^2–4^. Experimental studies using animal models have shown that ventricular pacing, which induces an asynchronous electrical activation and contraction pattern, produces both global and local LV remodeling in the form of ventricular dilation and asymmetrical hypertrophy, respectively^5^.

Computational modeling is increasingly used to simulate long-term changes associated with cardiac growth. Formulated based on the volumetric growth framework in which the deformation gradient tensor is multiplicatively decomposed into an elastic and a growth component^6,7^, cardiac growth constitutive models are developed to phenomenologically describe local changes in shape and size of the myocytes in response to local alterations of cardiac mechanics (i.e., stresses) and/or kinematics (i.e., strains). These constitutive models are usually coupled with a computational cardiac mechanics model to simulate the collective geometrical changes of the myocytes associated with alterations in loading conditions^8–11^ in a realistic ventricular geometry. Existing computational cardiac growth models have largely focused on describing pathologies associated with global alterations in loading conditions, such as pressure and volume overload that produce concentric and eccentric hypertrophy, respectively^9,10,12,13^. Except for one computational model^14^, little has been done to simulate long-term changes associated with alterations of the electrical conduction pattern in the heart. In that model, longitudinal and transverse growth of the myocyte are controlled by the normal strains in those respective directions. Recent experiment data^15^, however, has shown that stretching the myocyte longitudinally can produce both series and parallel addition of the sarcomeres that result in longitudinal and transverse growth, respectively.

Motivated by these experimental observations, we seek here to investigate if prescribing myofiber stretch as a single stimulus that controls growth in the myofiber and transverse directions (with different sensitivity) can quantitatively reproduce long term changes in ventricular geometry associated with mechanical dyssynchrony. Based on a recently developed modeling framework^8,16^, we simulate anisotropic growth that is driven solely by the local maximum elastic myofiber stretch. We seek to determine if it is possible to find different forward and reverse growth rates in the longitudinal and transverse directions that can simultaneously and quantitatively reproduce global and local asymmetrical changes in biventricular geometry associated with those found in previous chronic pacing experiments^5,17^. We show that with appropriate calibration of the growth rates, the coupled electromechanics-growth model can reproduce changes in the biventricular geometry that are qualitatively comparable with previous experimental measurements and clinical observations^5,17^, suggesting that it is possible for myofiber stretch to be the sole growth stimulant in mechanical dyssynchrony.

## Results

We present short-term and long-term results of anisotropic growth and remodeling (G&R) constitutive model. Details of the model and simulation cases are given in the Method section.

### Activation patterns

**Figure 1:**
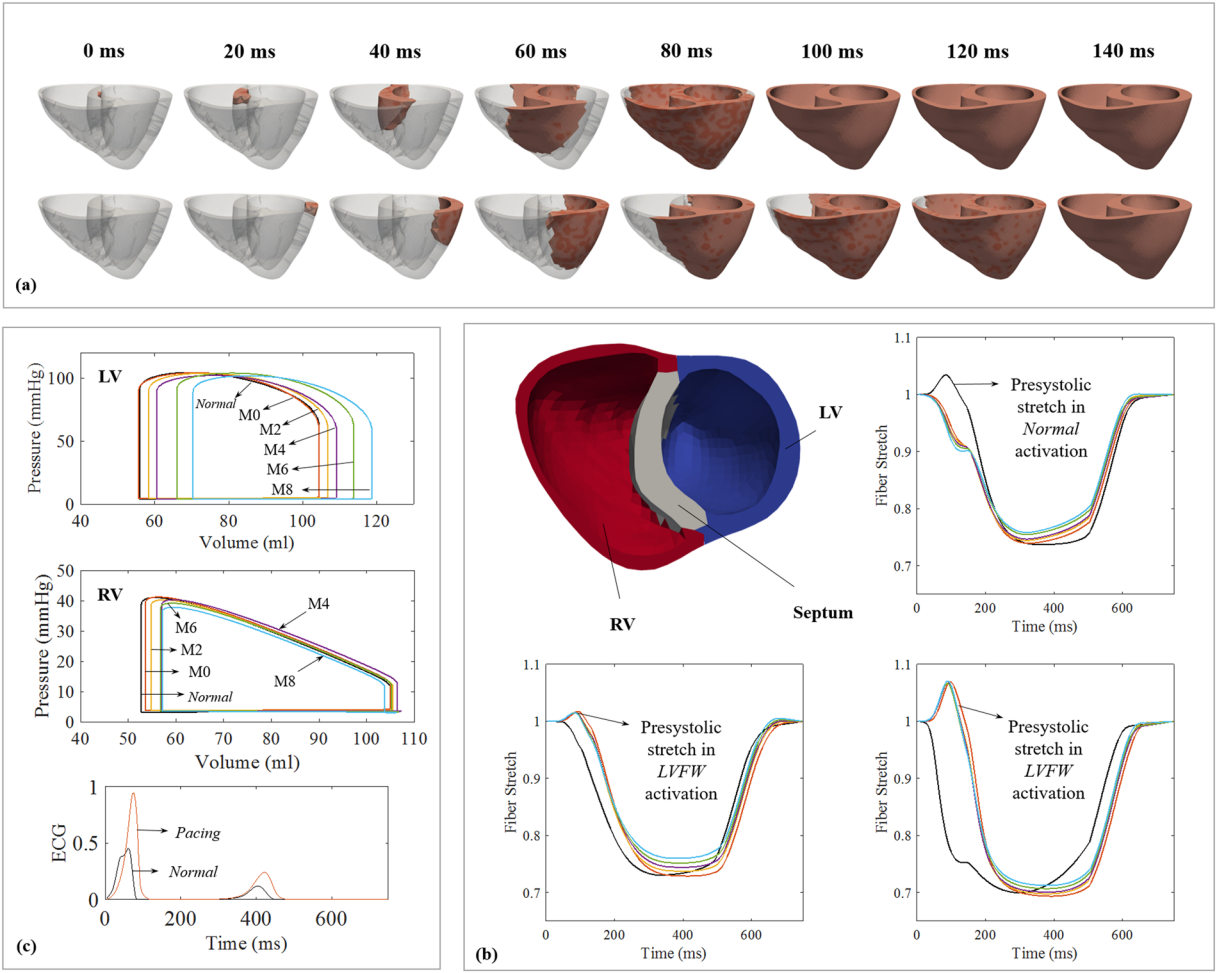
(**a**) Propagation of the depolarization isochrones in the Normal (top) and Pacing (bottom) cases. (**b**) Long term changes in RV (left) and LV (right) PV loops in the Pacing case; M0–8 denote results at 0–8 month. Refer to (**c**) for line color. (**c**) Fiber stretch *λ*_*f*_ as a function of time over a cardiac cycle at 0–8 month. Normal case is in black.

Figure 1a shows the activation patterns for the *Normal* and *Pacing* cases. In the *Normal* case, the depolarization isochrones propagated towards the left ventricular free wall (LVFW) and right ventricular free wall (RVFW) upon septal initiation. The entire biventricular unit was activated at about 80 ms (approximately the QRS duration). Conversely in the *Pacing case*, depolarization isochrones propagated from the LVFW towards the septum and then the RVFW, resulting in a longer activation time of ∼120 ms for the entire biventricular unit.

### Regional variation of myofiber stretch

Figure 1b shows the spatially-averaged elastic myofiber stretches *λ*_*f*_ in the RVFW, septum, and LVFW for the *Pacing* and *Normal* cases taken with respect to the end-diastolic configuration. In the *Pacing* case, pre-systolic myofiber stretch (i.e., *λ*_*f*_ > 1) was present only in the late-activated RVFW and septum, with a higher value found in the latter. By contrast, only a small pre-systolic myofiber stretch was found at the LVFW in the *Normal* case compared to that in the septum of the *Pacing* case. Consequently, myofiber stretch in the septum + RVFW and LVFW of the *Pacing case* deviated positively and negatively from the homeostatic value in the *Normal* case. This heterogeneity in myofiber stretch *λ*_*f*_ resulted in the evolution of growth scalars *θ*_*i*_’s towards *θ*_*i*,*max*_ in the late activated septum/RV, and *θ*_*i*,*max*_ in the early-activated LVFW, leading to long-term asymmetrical geometrical changes.

### Pressure-volume relationship

Figure 1c shows the long-term changes in the LV and right ventricle (RV) pressure-volume (PV) loops for the *Pacing* case. There was no immediate substantial reduction in pump function in the *Pacing* case (0 month). Changes were, however, noticeable at 2 months in the form of a progressive LV dilation. Specifically, in the span of 8 months, LV end-diastolic volume (EDV) increased from 104.3 ml to 118.6 ml whereas end-systolic volume (ESV) increased from 55.65 ml to 70.3 ml. This led to a rightward shift in the LV PV loop that was accompanied by a slight change in ejection fraction (EF) from 48.7% to 48.3% in 8 months. On the other hand, the simulations also show long-term changes of the RV PV loops due largely to thickening of the septum. RV EDV slightly decreased from 104.9 ml to 103.7 ml whereas RV ESV increased from 53.6 ml to 57.1 ml at 8 months.

### Long-term local geometrical changes

**Figure 2:**
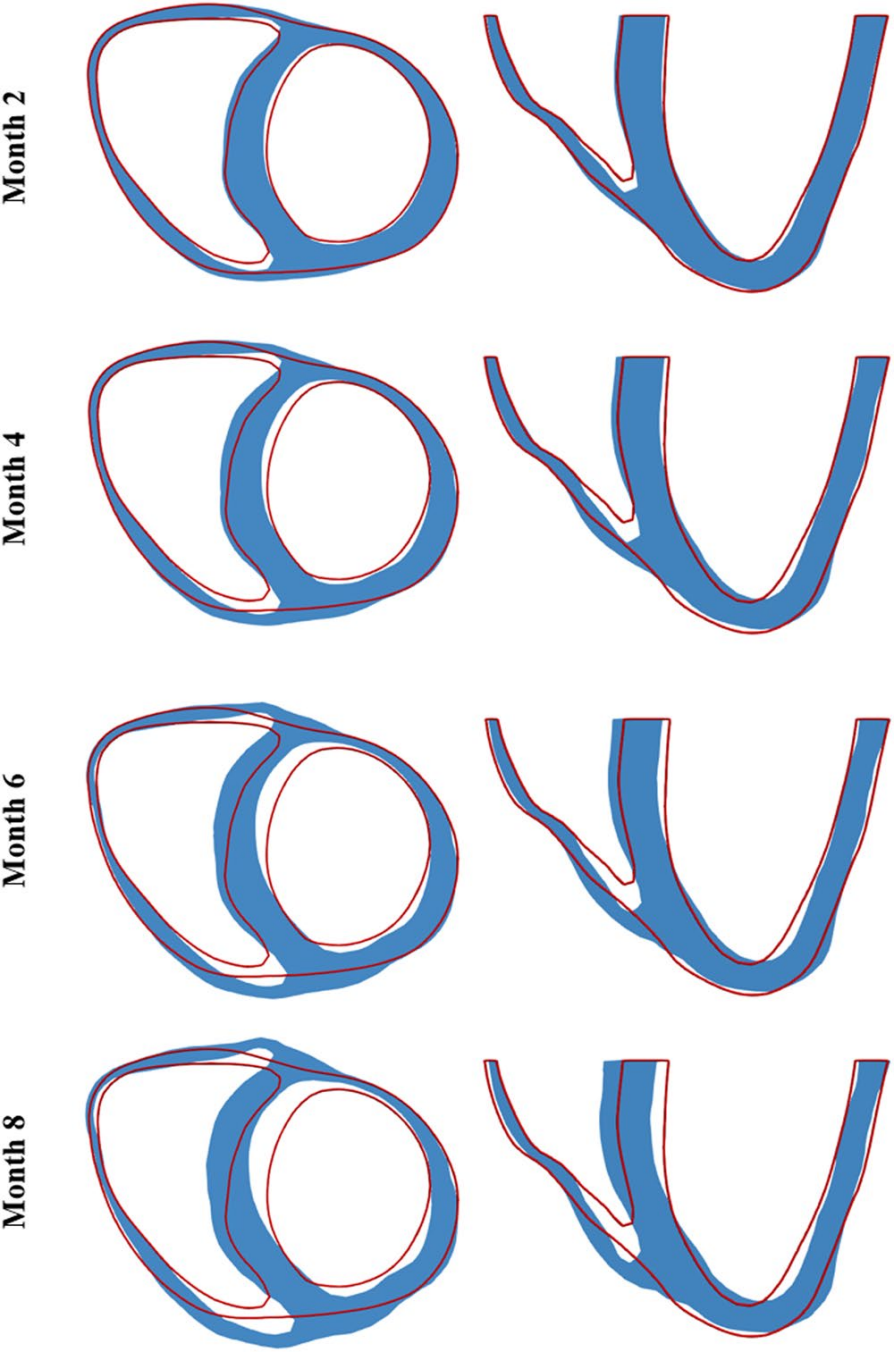
Long-term changes in biventricular geometry (blue) are superimposed on the original (red outline). Left: short-axis view; Right: long-axis view.

**Figure 3:**
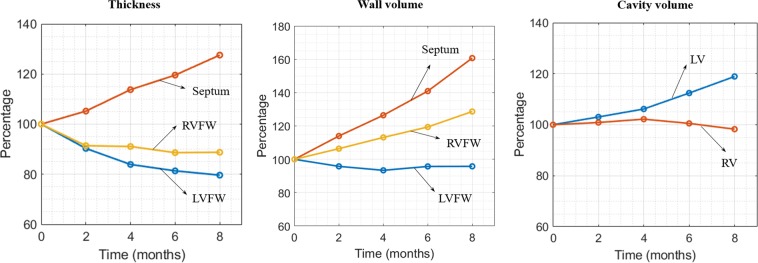
Long-term local geometrical changes. Left: wall thickness; Middle: wall volume; Right: Cavity volume.

Figure 2 shows the long-term changes in geometry of a short-axis section taken at the mid-ventricular level and a long-axis section that are typically assessed in experimental and clinical studies. As shown in the figure, long-term changes in the geometry is highly asymmetrical in the biventricular unit in the *Pacing* case with radial wall thickening occurring at the late-activated septum and wall thinning occurring at the early-activated LVFW. Shown quantitatively in Fig. 3, the model predicted a monotonic increase in LV EDV (13.7%), septum wall volume (60.7%) and septum wall thickness (27.5%) over an 8 months period. Conversely, RVFW and LVFW thickness decreased by (12.3%) and (20.4%) over the same period, respectively. Wall volume of the LVFW and RVFW as well as RV EDV behaves non-monotonically and were roughly unchanged after 8 months.

## Discussion

Motivated by a recent experiment showing that longitudinal stretching of myocyte produces both longitudinal and transverse growth via parallel and serial sarcomeric addition^15^, we have presented an anisotropic G&R constitutive model in which the changes in lengths of the tissue in 3 orthogonal material directions are driven locally by the deviation of maximum elastic myofiber stretch (over a cardiac cycle) from its corresponding homeostatic set point value. This model is coupled to an electromechanics modeling framework to simulate the long-term effects of asynchronous activation associated with LVFW pacing. We find that the application of elastic myofiber stretch as the sole mechanical driver of G&R in the constitutive model can reproduce quantitatively both local and global features of asymmetrical remodeling found in experiments of mechanical dyssynchrony as presented below.

In terms of acute behavior, our simulations show that in the presence of electromechanics alterations induced by LVFW pacing, a pre-stretch occurs at the late activated regions (Septum + RVFW) in the beginning of systole (Fig. 1) that resulted in a higher maximum elastic myofiber stretch compared to the homeostatic set point value (during normal activation) found in those regions. The early activated region (LVFW), on the other hand, has a lower maximum elastic myofiber stretch when compared to its corresponding homeostatic set point value. These results are consistent with observations made in animal models of asynchronous activation^18,19^ and LBBB patients^20^, where abnormal stretching of the tissue at the beginning of systole (i.e., pre-systolic stretching) were found at the late activated regions.

Using the alteration of elastic myofiber stretch as a stimulant for G&R in all 3 material directions, the model predictions, after appropriate calibration of parameters, are compared favorably to local and global measurements in canine experiments with similar chronic LVFW pacing protocol^5,17^. A quantitative comparison of the experimental results with model predictions are given in Table 1. In terms of local geometrical changes, the model predicted an increase in septum wall thickness by 18.5% (cf. 23 ± 12% in the experiments^5^) and a decrease in LVFW thickness by 19.7% (cf. 17 ± 17% in the experiments^5^) after 6 months of pacing. In terms of global geometrical changes, the model predicts an increase in LV EDV by 9% (cf. 7.4 ± 29% in the experiments) and LVFW + Septum wall volume by 9.5% (cf. 15 ± 17% in the experiments) for the same duration. As detailed in Table 1, the chronic features of LVFW pacing predicted by the model are also found in LBBB^21^, which produces mechanical dyssynchrony via an opposite activation pattern (i.e., septum is activated first followed by the LVFW).

**Table 1:** Comparison with experimental data^5,17,21^.

Parameter	Month	LVFW pacing Simulation	LVFW Pacing Experiment (Van Oosterhout *et al*., 1998)	LBBB Experiment (Vernooy *et al*., 2004)	LVFW Pacing Experiment (Prinzen *et al*., 1995)
LVEDV (% of Normal”)	0	100	100 ± 27.8	100 ± 29.8	100
	2	102.3	—	117.5 ± 12.8	—
	4	104.6	—	129.8 ± 50.9^R^	—
	6	109	107.4 ± 29.9^M^	—	—
	8	113.7	—	—	—
LVEF (%)	0	48.7	35.3 ± 7.0	43 ± 4	100
	2	48.3	—	—	—
	4	48.6	—	33 ± 6^R^	—
	6	47.8	39.6 ± 8.9^M^	—	—
	8	48.3	—	—	—
LVESV (% Change)	0	100	100	—	100
	2	104.9	—	—	—
	4	108.7	—	—	—
	6	118.5	—	—	—
	8	126.4	—	—	—
Early activated region thickness (% change)	0	100^L^	100^L^	100^C^	100^L^
	2	90.3^L^	90.7 ± 8.7^L^	—	88.9 ± 6.8^L,R^
	4	83.9^L^	87.0 ± 7.2^L^	—	79.7 ± 8.0^L,R,*^
	6	81.3^L^	86.5 ± 16.7^L,R^	—	—
	8	79.6^L^	—	—	—
Late activated region thickness (% Change)	0	100^C^	100^C^	100^L^	100^C^
	2	105.2^C^	108.4 ± 11.3^C^	—	96.8^C,M^
	4	113.7^C^	110.5 ± 16.8^C^	—	103.0 ± 7.5^C,M,*^
	6	119.6^C^	122.5 ± 11.3^C,R^	—	—
	8	127.5^C^	—	—	—
RV thickness (% Change)	0	100	100	100	100
	2	91.4	—	—	—
	4	91.1	—	—	—
	6	88.6	—	—	—
	8	88.7	—	—	—

^M^denotes no significant change over time; ^R^denotes significant change over time (p < 0.05).

^L^Denotes LVFW Thickness; ^C^denotes septum thickness.

^*^Denotes 3 months; ^—^denotes not reported or measured.

Interestingly, our model also predicted that the RV wall thickness is changed in LVFW pacing in response to the presence of a pre-systolic stretch (Fig. 1). Unfortunately, measurements of RVFW geometrical changes in response to LVFW pacing are, to the best of our understanding, not available for comparison. While there are a small number of studies on right branch bundle block (RBBB) which has similar (left to right) activation pattern as in LVFW pacing, the presence of RBBB is either superimposed on tachypacing-induced heart failure^22^ or is confounded by other diseases such as Tetralogy of Fallot^23^.

Our study shows that it is necessary to impose different forward growth rate *τ*_*g*_ and reverse growth rate *τ*_*rg*_ in order to reproduce asymmetrical hypertrophy. Additional simulations using the same forward and reverse growth rates produce excessive thickening in the septum compared to LVFW case and are not able to predict the observed asymmetrical hypertrophy (See Appendix). While direct measurements of reverse growth rates on isolated cardiomyocytes are, to the best of our knowledge, not available, large animal and clinical studies have suggested that the reverse growth rates are likely to be different from the forward growth rates. For example, the study by Vernooy *et al*.^24^ found that regional LV mass does not return to normal values (100%) during biventricular pacing. Similarly, the study by Gerdes *et al*.^25^ also suggests that cardiac hypertrophy induced by aortocaval fistula in rats may not be completely reversible upon reversal of fistula.

As with most constitutive models describing cardiac G&R, the model described here is developed based on the volumetric growth framework^6^ that largely focuses on changes in the morphology of the myocytes as they undergo hypertrophy or atrophy (as opposed to turnover of extracellular matrix constituents) during G&R^11^. One of the key differences between these G&R constitutive models is in the prescription of G&R stimuli in their formulation. Goktepe^9^ *et al*. proposed using two different types of G&R stimuli, namely, elastic myofiber stretch for driving growth in the myofiber direction and Mandel stress for driving growth in directions transverse to the myofiber. They show that this formulation can reproduce features that are qualitatively in agreement with eccentric and concentric hypertrophy (i.e., volume and pressure overload). It is not clear, however, if it can predict asymmetrical remodeling features found in mechanical dyssynchrony. Subsequently, Kerckhoffs *et al*.^10^ proposed using a single type of G&R stimuli, namely, strain in their model. While the G&R stimuli in the myofiber direction is similar to Goktepe *et al*.^9^ in their model, they proposed using normal strain components transverse to the myofiber direction as stimuli for growth in the transverse directions. They showed that this formulation is able to reproduce features associated with global remodeling in pressure and volume overload as well as local remodeling associated with LBBB^14^. A detailed comparison of existing cardiac G&R constitutive models based on equibiaxial loading can be found in Witzenburg and Holmes^26^. In contrast to the G&R models described above, elastic myofiber stretch is prescribed here as the driver for growth in both the myofiber and transverse directions, but with different sensitivity (or rates). This assumption is consistent with a recent study that observed in real time both longitudinal elongation and lateral extension occurring in the myocyte in response to longitudinal stretching^15^. With appropriate calibration, we show that the prescription of a single growth stimuli based only on elastic myofiber stretch can quantitatively reproduce the largely local G&R features that are associated with mechanical dyssynchrony, which reinforce the theory that transverse growth maybe controlled, at least to some extent, by elastic myofiber stretch that previous models have not considered.

Although the anisotropic growth model predictions are generally consistent with experimental and clinical measurements, the model presented here, nonetheless, could be further improved with time. *First*, we have not considered sarcomeric parallel addition under lateral stretch as found in the experiment^15^ because changes in myofiber (longitudinal) stretch is the most prominent asymmetrical feature of asynchronous activation. Moreover, without any direct measurements of forward and reverse growth rates associated individually with lateral and longitudinal stretch, it is difficult to separate parallel addition of sarcomere due to changes in lateral stretch from that due to longitudinal stretch to reproduce clinically observed features of asynchronous activation. Inclusion of lateral stretch as a growth stimuli in the model can be incorporated in future studies with the availability of more experimental data. *Second*, we did not consider any myocardial material property changes that may occur during the remodeling process. This limitation is shared by growth models developed based purely on the volumetric growth framework, which cannot directly accommodate for any changes in the mechanical properties. Structural changes in the tissue such as the reorientation of the collagen fibers were also not considered in our simulations. Nonetheless, the experiments did not report any change in the myocardial collagen fraction^5^, suggesting that extracelluar matrix remodeling is limited in mechanical dyssynchrony. *Third*, we have also assumed a constant preload and afterload in the entire simulation. Besides ventricular G&R, the circulatory system may also adapt during the long-term^27^. Specifically, models reflecting improvements in ventricular-arterial coupling during reverse remodeling^28^ are expected to better capture long-term changes in CRT. We also did not account for any electrophysiological remodeling that may occur during electromechanics alterations. Besides changes in geometry, the myocyte’s electrophysiological properties such as action potential duration^29^ may be altered during the remodeling process. *Last*, our simulations did not consider the fact that asynchronous contraction can induce mitral regurgitation (MR), which may in turn confound the G&R process by inducing volume overload. Evidence that asynchronous contraction produces MR is supported by the observation that the severity of baseline MR is decreased in CRT patients^30,31^ when their mechanical dyssynchrony is corrected. The model, which we have shown is able to reproduce features associated with volume (and pressure) overload (See Appendix) using fiber stretch as the growth stimuli, can be used in future studies to evaluate such possibilities.

In summary, we have presented an anisotropic growth model that simulates asymmetric growth in mechanical dyssynchrony. We show that by using the maximum elastic fiber stretch as the sole growth stimulant, the model can reproduce long-term features that agree quantitatively with the experimental and clinical findings. The growth model may be useful for optimizing the long-term pacing effects of cardiac resynchronization therapy in future studies.

## Methods

### Growth constitutive model

**Figure 4:**
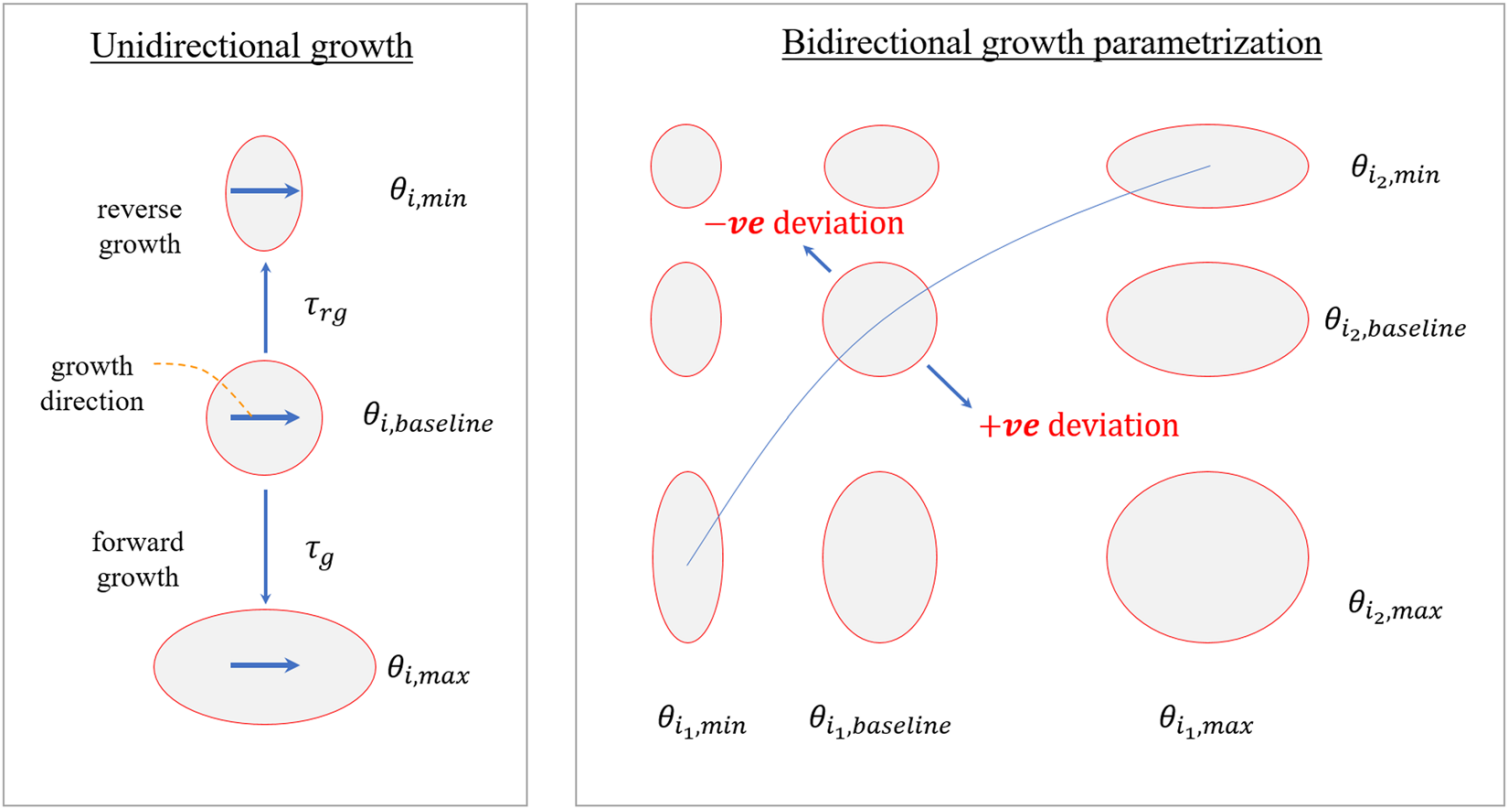
Schematic of the anisotropic growth evolution in response to local stimulus.

Here we let ${\chi }_{{\kappa }_{0}}({\boldsymbol{X}},\,t)$ describe the mapping from an unloaded (stress-free) reference configuration *κ*_0_ with material point position ***X*** to a current configuration *κ* with the corresponding material point position ${\boldsymbol{x}}={\chi }_{{\kappa }_{0}}({\boldsymbol{X}},\,t)$. The displacement field is given by ***u***(***X***) = ***x*** − ***X*** and the deformation gradient tensor corresponding to the mapping is defined as ${\boldsymbol{F}}=\frac{\partial {\chi }_{{\kappa }_{0}}}{\partial {\boldsymbol{X}}}$. Under the volumetric growth framework^6^, we multiplicatively decomposed the deformation gradient tensor, i.e.

1 $${\boldsymbol{F}}={{\boldsymbol{F}}}_{e}{{\boldsymbol{F}}}_{g},$$

into an elastic component ***F***_***e***_ and an incompatible growth component ***F***_***g***_. The growth deformation gradient ***F***_***g***_ was parameterized by the scalar growth multipliers *θ*_*f*_, *θ*_*s*_ and *θ*_*n*_ in the following tensorial form

2 $${{\boldsymbol{F}}}_{g}=\,{\theta }_{f}\,{{\boldsymbol{f}}}_{0}\otimes {{\boldsymbol{f}}}_{0}+\,{\theta }_{s}\,{{\boldsymbol{s}}}_{0}\otimes {{\boldsymbol{s}}}_{0}+{\theta }_{n}\,{{\boldsymbol{n}}}_{0}\otimes {{\boldsymbol{n}}}_{0},$$

where ***f***_0_, ***s***_0_ and ***n***_0_ are local myofiber, sheet, and sheet-normal directions in the reference configuration. The evolution of the growth multipliers was described based on deviations of a prescribed stimulant *s*_*j*_ from its homeostatic value *s*_*i*,*h*_. Specifically, the evolution of growth multipliers was described by the following ordinary differential equation^16^.

3 $${\dot{\theta }}_{i}={k}_{i}({\theta }_{i},\,{s}_{i}){g}_{i}({s}_{i}-{s}_{i,h})\,{\rm{for}}\,i=f,\,s,\,n,$$

where (^.^) is the derivative with respect to time *t* and *k*_*i*_ (*θ*_*i*_, *s*_*i*_) are rate limiting functions that restrict forward and reverse growth rates. The function *g*_*i*_ (*s*_*i*_− *s*_*i*,*h*_) depends on local stimulus and we prescribed a simple linear form, i.e., *g*_*i*_ (*s*_*i*_ − *s*_*i*,*h*_) = *s*_*i*_ − *s*_*i*,*h*_. The rate limiting function was defined as follows

4 $${k}_{i}({\theta }_{i},\,{s}_{i})=\{\begin{array}{c}\frac{1}{{\tau }_{g,i}}{(\frac{{\theta }_{max,i}-{\theta }_{i}}{{\theta }_{max,i}-{\theta }_{min,i}})}^{{\gamma }_{g,i}}{\rm{if}}\,{g}_{i}({s}_{i}-{s}_{i,h})\ge 0\\ \frac{1}{{\tau }_{rg,i}}{(\frac{{\theta }_{i}-{\theta }_{min,i}}{{\theta }_{max,i}-{\theta }_{min,i}})}^{{\gamma }_{rg,i}}{\rm{if}}\,{g}_{i}({s}_{i}-{s}_{i,h}) < 0\end{array},$$

where the subscript *i* = *f*, *s*, *n* denote the association with the myofiber, sheet, and sheet-normal directions. The growth constitutive model parameters *τ*_*g*,*i*_, *τ*_*rg*,*i*_, *γ*_*g*,*i*_ and *γ*_*rg*,*i*_ control the rates of growth and reverse growth, which may be different. The main purpose of the rate-limiting function is to restrict the evolution of the growth multipliers *θ*_*i*_ to lie within some prescribed limits^7^ (Fig. 4). Allowing the rate limiting function *k*_*i*_ (*θ*_*I*_, *s*) to be prescribed individually in each *i* direction enables a broad spectrum of anisotropic growth deformation to be produced.

Based on previous experimental findings^15^, maximum elastic myofiber stretch was prescribed as the growth stimuli in all 3 material directions (i.e., *s*_*i*_ = *λ*_*f*_ for *i* = *f*, *s*, *n*). The myofiber stretch was defined by

5 $${\lambda }_{f}=\sqrt{{{\boldsymbol{f}}}_{0}.{{\boldsymbol{C}}}_{ED}{{\boldsymbol{f}}}_{0}},$$

where ***C***_*ED*_ denotes the right Cauchy-Green deformation tensor with respect to the end-diastolic configuration that was, in turn, defined by ${{\boldsymbol{C}}}_{ED}={{\boldsymbol{F}}}_{ED}^{T}{{\boldsymbol{F}}}_{ED}$ with ***F***_*ED*_ = ***F***_*e*_(***F***_*r*,*ED*_)^−1^ and ***F***_*r*_,_ED_ representing the map from the unloaded configuration to the end-diastolic configuration. As depicted in Fig. 4, positive deviations from homeostatic stretch in the growth constitutive model will result in the growth multiplier *θ*_*i*_ evolving towards *θ*_*max*_,_*i*_, which is typically an expanded configuration. Conversely, negative deviations from homeostatic stretch will result in the growth multiplier *θ*_*i*_ evolving towards *θ*_*min*_,_*i*_, which is typically a shrunk configuration.

### Electromechanics model

#### Cardiac electrophysiology

Cardiac electrical activity and its propagation was modeled using the modified Fitzhugh-Nagumo and monodomain equations^32^. Specifically, the spatio-temporal evolution of cardiac action potential $\dot{\varphi }$ was described in the reference configurations by

6a $$\dot{\varphi }=div({\boldsymbol{D}}\,grad\,\varphi )+{f}_{\varphi }(\varphi ,r)+{I}_{s},$$

6b $$\dot{r}={f}_{r}(\varphi ,r),$$

where ${\boldsymbol{D}}={d}_{iso}{\boldsymbol{I}}+{d}_{ani}{{\boldsymbol{f}}}_{0}\otimes {{\boldsymbol{f}}}_{0}$ is the anisotropic electrical conductivity tensor (with faster conduction along myofiber direction), *I*_*s*_ is the constant electrical stimulus for prescribing local excitation initiation and pacing or both. Variables *ϕ* and *r* are dimensionless and *r* is a recovery variable. The excitation properties of cardiac tissue were defined using:

7a $${f}_{\varphi }=c\varphi (\varphi -\alpha )(1-\varphi )-r\varphi ,$$

7b $${f}_{r}=(\gamma +r\bar{\gamma }(\varphi ))(-r\,c\varphi (\varphi -b-1)),$$

which is a modification of the Fitzhugh-Nagumo equations^33,34^ following Aliev and Panfilov^32^ with model parameters *c*, *α*, *b*, and *γ*. The function $\bar{\gamma }(\varphi )={\mu }_{1}/({\mu }_{2}+\varphi )$ in Eq. (7b) allows for the tuning of cardiac restitution with parameters *μ*_1_ and*μ*_2_.

#### Cardiac mechanics

An active stress formulation was used to describe the mechanical behavior of the cardiac tissue. In this formulation, the cardiac tissue constitutive relation was prescribed separately for passive and active mechanical behavior. Specifically, the second Piola-Kirchkoff or PK2 stress tensor ***S*** was additively decomposed into a passive component ***S***_*p*_ and an active component***S***_*a*_, i.e.:

8 $${\boldsymbol{S}}={{\boldsymbol{S}}}_{p}+{{\boldsymbol{S}}}_{a}.$$

The tissue passive mechanical behavior was described using the following Fung-type^35^ strain energy function based on the Green-Lagrange elastic strain tensor ***E***_*e*_:

9 $$W({{\boldsymbol{E}}}_{e})=\frac{C}{2}(\exp (Q)-1),$$

where $Q={b}_{f}{E}_{ff}^{2}+{b}_{fs}({E}_{fs}^{2}+{E}_{sf}^{2}+{E}_{fn}^{2}+{E}_{nf}^{2})+{b}_{xx}({E}_{ss}^{2}+{E}_{nn}^{2}+{E}_{ns}^{2}+{E}_{sn}^{2})$ with material parameters *C*, *b*_*f*_, *b*_*fs*_, *b*_*xx*_, and *E*_*ij*_ with (*i*,*j*) ∈ (*f*, *s*, *n*) denoting the components of the elastic Green-Lagrange strain tensor ${{\boldsymbol{E}}}_{e}=\frac{1}{2}({{\boldsymbol{F}}}_{e}^{T}{{\boldsymbol{F}}}_{e}-1)\,$corresponding to the material coordinates. Incompressibility of the tissue was enforced by an augmented strain energy function

10 $$\hat{W}({{\boldsymbol{E}}}_{e},\,p)=W({{\boldsymbol{E}}}_{e})-p(J-1),$$

where *p* is a Lagrange multiplier for enforcing the kinematic constraint $J=\det \,{{\boldsymbol{F}}}_{e}=1$. The stress tensor was derived from the augmented strain energy function by

11 $${{\boldsymbol{S}}}_{p}=\frac{\partial \hat{W}({{\boldsymbol{E}}}_{e},\,p)}{\partial {{\boldsymbol{E}}}_{e}}.$$

A phenomenological active contraction model was used to describe contraction of the tissue upon excitation. In this model, the active (fiber-directed) stress tensor was defined by:

12 $${{\boldsymbol{S}}}_{a}=T(t,\,{t}_{init}({\boldsymbol{X}}),\,{C}_{a0},\,{E}_{ff},\,{T}_{max}){{\boldsymbol{f}}}_{0}\otimes {{\boldsymbol{f}}}_{0}.$$

Magnitude of active force *T* was defined by a sarcomere length-dependent contractile force generation model^36,37:^

13 $$T(t,\,{t}_{init}({\boldsymbol{X}}),\,{C}_{a0},\,{E}_{ff},\,{T}_{max})={T}_{max}\tfrac{{C}_{a0}^{2}}{1+E{C}_{a50}^{2}({E}_{ff})}\tfrac{1-\,\cos (\omega (t,\,{t}_{init},\,{E}_{ff}))}{2}$$

where *C*_*a*0_ is the peak intracellular calcium concentration, *T*_*max*_ is a scaling factor for the tissue contractility, and *EC*_*a*50_ is the length-dependent calcium sensitivity given by:

14 $$E{C}_{a50}=\frac{{({C}_{a0})}_{max}}{\sqrt{\exp (B(l-{l}_{0}))-1}}$$

with material constant *B*, prescribed maximum peak intracellular calcium concentration (*C*_*a*0_)_*max*_, instantaneous sarcomere length *l*, and sarcomere length at which no active tension develops *l*_0_. The instantaneous sarcomere length was defined based on the prescribed initial length of sarcomere*l*_*s*0_, and was calculated using $l={l}_{s0}\sqrt{{{\boldsymbol{f}}}_{0}.{{\boldsymbol{C}}}_{e}{{\boldsymbol{f}}}_{0}\,}$.

### Coupling electrophysiology and mechanics

The active contraction model was modified to incorporate a spatially heterogeneous activation initiation time *t*_*init*_(***X***) that accounts for asynchronous depolarization based on the action potential *ϕ*. The function *ω* in Eq. (13) is given by:

15 $$\omega =\{\begin{array}{ll}\pi \frac{{t}_{sa}}{{t}_{0}} & {\rm{if}}\,0\le {t}_{sa} < {t}_{0}\\ \pi \frac{{t}_{sa}-{t}_{0}+{t}_{r}}{{t}_{r}} & {\rm{if}}\,{t}_{0}\le {t}_{sa} < {t}_{0}+{t}_{r}\\ 0 & {\rm{if}}\,{t}_{0}\le {t}_{sa} < {t}_{0}+{t}_{r}\end{array}$$

that explicitly defines active contraction based on time since activation *t*_*sa*_ (***X***) = *t*_*current*_− *t*_*init*_ (***X***), with *t*_*current*_ denoting the current time in the cardiac cycle as:

16 $${t}_{init}({\boldsymbol{X}})=\inf \{\,t({\boldsymbol{X}})|\varphi ({\boldsymbol{X}},t)\ge 0.9\}$$

defining the local initiation time that couples cardiac electrophysiology and mechanics. In Eq. (15), *t*_0_ is the prescribed time to maximum active tension and *t*_r_ is the sarcomere length-dependent active tension relaxation time that is given by *t*_*r*_ = *ml* + *b* with parameters *m* and *b*.

### Computational approximation

Discretizing time using an implicit backward Euler scheme, approximate solutions of the weak formulation for the acute electromechanics problem were obtained using the finite element (FE) method, where we seek to find ***u*** ∈ ***H***^1^(Ω), *p* ∈ *L*^2^(Ω), *P*_*LV*_ ∈ ${\mathbb{R}}$, *P*_*RV*_ ∈ ${\mathbb{R}}$, ***c***_1_ ∈ ${{\mathbb{R}}}^{3}$, ***c***_2_ ∈ ${{\mathbb{R}}}^{3}$, *φ* ∈ ***H***^1^(Ω), *r* ∈ ***H***^0^(Ω), such that the below equations:

17a $$\begin{array}{rcl}\delta  {\mathcal L}  & = & {\int }_{\Omega }({\boldsymbol{FS}}-J{{\boldsymbol{F}}}^{-T}):grad\,\delta {\boldsymbol{u}}\,dV-{\int }_{\Omega }\delta p(J-1)dV\\  &  & -{P}_{LV}{\int }_{{\Omega }_{LV,cav}}J{{\boldsymbol{F}}}^{-T}:grad\,\delta {\boldsymbol{u}}\,dV-{P}_{RV}{\int }_{{\Omega }_{RV,cav}}J{{\boldsymbol{F}}}^{-T}:grad\,\delta {\boldsymbol{u}}\,dV\\  &  & -\delta {P}_{LV}({V}_{LV,cav}({\boldsymbol{u}})-{V}_{LV,p})-\delta {P}_{RV}({V}_{RV,cav}({\boldsymbol{u}}){V}_{RV,p})\\  &  & -\delta {{\boldsymbol{c}}}_{1}.{\int }_{\Omega }{\boldsymbol{u}}dV-\delta {{\boldsymbol{c}}}_{2}.{\int }_{\Omega }{\boldsymbol{X}}\times {\boldsymbol{u}}\,dV-{{\boldsymbol{c}}}_{1}.{\int }_{\Omega }\delta {\boldsymbol{u}}dV-{{\boldsymbol{c}}}_{2}.{\int }_{\Omega }{\boldsymbol{X}}\times \delta {\boldsymbol{u}}dV\\  &  & -{\int }_{\partial {\Omega }_{epi}}{k}_{spring1}{\boldsymbol{u}}.\delta {\boldsymbol{u}}dS-{\int }_{\partial {\Omega }_{b}}{k}_{spring2}{\boldsymbol{u}}.\delta {\boldsymbol{u}}dS=0\end{array}$$

17b $${\int }_{\Omega }(\varphi -{\varphi }_{n}){\rm{\Delta }}{t}^{-1}\delta \varphi ={\int }_{\Omega }{\boldsymbol{D}}\,grad\,\varphi .\,grad\,\delta \varphi +{\int }_{\Omega }({f}_{\varphi }+{I}_{s})\,\delta \varphi $$

17c $${\int }_{\Omega }(r-\,{r}_{n}){\rm{\Delta }}{t}^{-1}\delta r={\int }_{\Omega }{f}_{r}\,\delta \varphi $$

are satisfied for all test functions *δ****u*** ∈ ***H***^1^(Ω), *δp* ∈ *L*^2^(Ω), *δP*_*LV*_ ∈ ${\mathbb{R}}$, *δP*_*RV*_ ∈ ${\mathbb{R}}$, *δ****c***_1_ ∈ ${{\mathbb{R}}}^{3}$, *δ****c***_2_ ∈ ${{\mathbb{R}}}^{3}$, *δφ* ∈ ***H***^1^(Ω), *δr* ∈ ***H***^0^(Ω). Domains *Ω*_*LV*,*cav*_ and *Ω*_*RV*,*cav*_ denote LV and RV cavities, respectively. Spring constants *k*_*spring*1_ and *k*_*spring*2_ model are associated with the robin-type boundary conditions imposed at the epicardial surface *∂Ω*_epi_ and basal surface *∂Ω*_b_, respectively. Variables ***c***_1_ and ***c***_2_ are Lagrange multipliers^38^ to constrain rigid body translation and rotation, respectively. Variables *P*_*LV*_ and *P*_*RV*_ are Lagrange multipliers^39^ constraining the LV and RV cavity volumes to their corresponding prescribed values *V*_*LV*,*p*_ and *V*_*RV*,*p*_, respectively. Cavity volumes *V*_*LV*,*cav*_ (***u***) and *V*_*RV*,*cav*_ (***u***) were estimated using the divergence theorem^39^. It can be shown that the computed Lagrange multipliers *P*_*LV*_ and *P*_*RV*_ are equivalent to the LV and RV cavity pressures, respectively. Note that *P*_*LV*_ and *P*_*RV*_ are different from the Lagrange multiplier *p* which is a scalar field, i.e., *p* = p(***C***, *t*) to enforce local incompressibility and depends on the spatial location of the myocardium.

We used a Galerkin FE discretization with piecewise quadratic element for the spatial approximation of displacements ***u***(***X***), continuous piecewise linear elements for hydrostatic pressure *p* and action potential *ϕ*. We used discontinuous Galerkin elements to approximate the recovery state variable *r* and fiber stretch *λ*_*f*_. A realistic human biventricular geometry, that was reconstructed from magnetic resonance images from a previous study^40^, was used for the computational study. Helix angle defining the myofiber direction ***f***_0_ was prescribed to vary linearly across the myocardial wall from 60° at the endocardium to −60° at the epicardium using a Laplace-Dirichlet rule-based algorithm^41^. Fiber directions ***f***_0_ as well as the orthogonal sheet direction ***s***_0_ and sheet-normal direction ***n***_0_ were prescribed at the quadrature points. A FE mesh with 11988 tetrahedral elements was used to discretize the biventricular geometry and FEniCS^42^ was used for computational implementation. The time interval (0, *T*) was discretized into subintervals[*t*_*n*_, *t*_*n* + 1_], *n* = 0, 1, …, with *t*_*n*_ = *n*Δ*t* and Δ*t* denoting a fixed time step.

#### Simulating the full cardiac cycle

**Figure 5:**
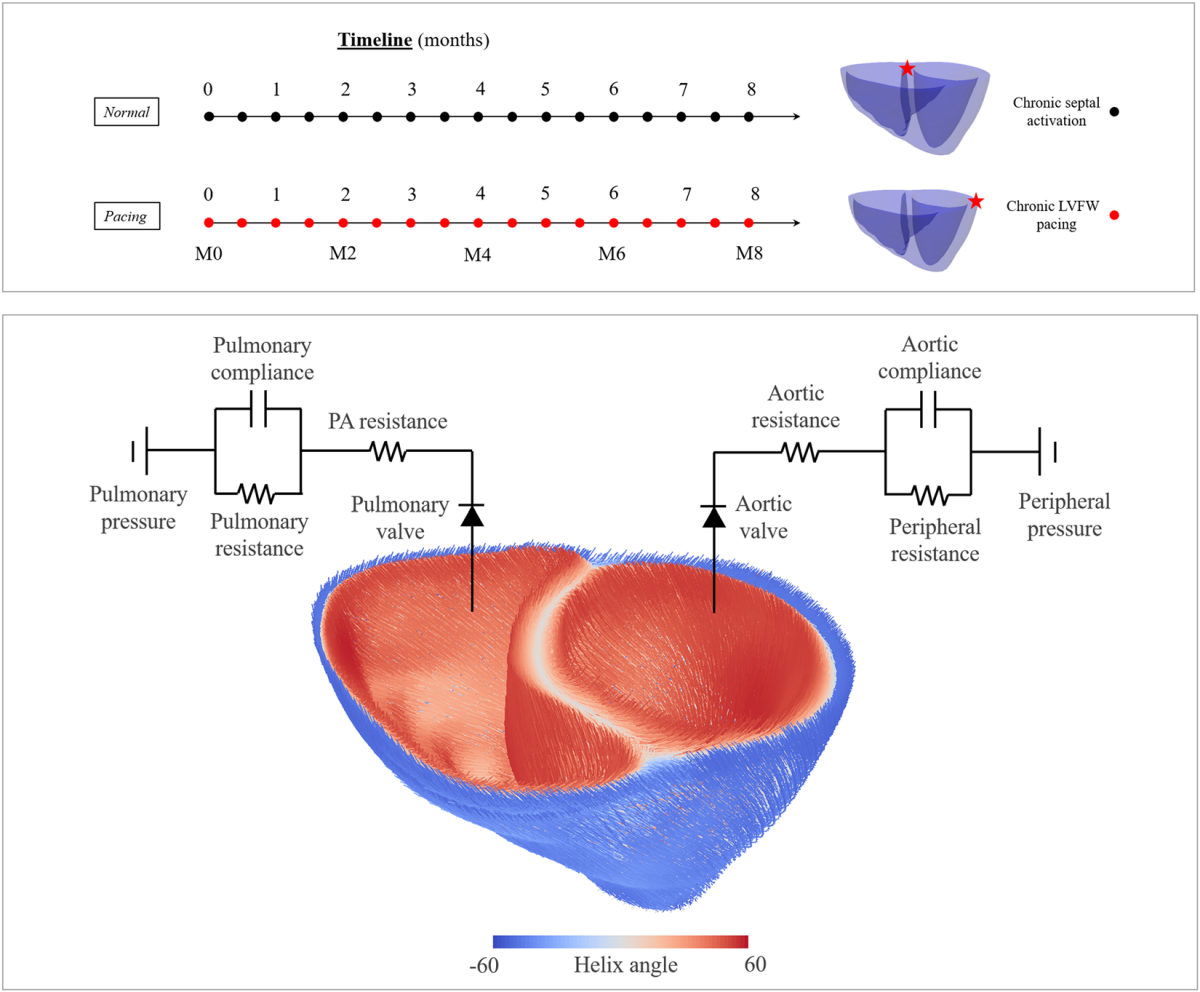
Top: Simulated chronic pacing timelines are shown. Pacing locations are indicated in the geometry using a red star. Bottom: Myofiber architecture and lumped circulation model.

Complete cardiac cycles with each having a duration of 750 ms were simulated using the electromechanics model. Specifically, passive filling was simulated by incrementally applying pressure to the LV and RV endocardial surfaces until their prescribed EDV was reached. Thereafter, systole was simulated by applying a current to different locations in the biventricular unit depending on the pacing protocol. The LV and RV cavity volumes were constrained to remain constant during the isovolumic contraction phase, which terminated when the LV and RV cavity pressures exceeded the prescribed aortic and pulmonary artery pressures, respectively. The subsequent ejection phase was simulated by coupling the LV and RV cavity volumes each to a 3-element Windkessel model (Fig. 5). Ejection phase in each cavity was terminated when the cavity outflow rate became less than 0.005 ml/min. The cavity volumes were then prescribed to remain constant to describe the isovolumic relaxation phase. The isovolumic relaxation phase was terminated based on prescribed pressures for initiation of diastolic filling, which was then passively prescribed until end of diastole.

### Separation of time scale between growth and elastic deformation

To integrate growth with the electromechanics model, we adopted a similar approach to separate the time scale as described in Lee *et al*.^8^. Specifically, since appreciable G&R can only be observed after large number of heartbeats, the growth tensor ***F***_*g*_ was treated as a constant that is independent of time in the electromechanics model and updated after each cardiac cycle. Approximate solutions of the weak formulation for the G&R problem were obtained using the FE method where we seek to find ***u*** ∈ ***H***^1^(Ω), *p* ∈ *L*^2^(Ω), such that the below equation

18 $$\begin{array}{rcl}\delta { {\mathcal L} }_{G} & = & {\int }_{\Omega }({\boldsymbol{FS}}-J{{\boldsymbol{F}}}^{-T}):grad\,\delta {\boldsymbol{u}}\,dV-{\int }_{\Omega }\delta p(J-1)dV\\  &  & -{\int }_{\partial {\Omega }_{epi}}{k}_{spring1}{\boldsymbol{u}}.\delta {\boldsymbol{u}}dS=0,\end{array}$$

is satisfied for all test functions for all *δ****u*** ∈ ***H***^1^(Ω) and *δp* ∈ *L*^2^(Ω). The above equation describing the long-term evolution differs from the Eq. (17) describing short-term evolution in the following ways: there is no active stress in the long-term model; and the short-term model was solved for cavity pressures with prescribed volumes while the long-term model is solved for cavity volumes at prescribed (zero) cavity pressures. We used a Galerkin FE discretization with piecewise quadratic FE for the spatial approximation of displacements ***u***(***X***). We used discontinuous Galerkin elements to approximate the growth parameters *θ*_*i*_s. The algorithm used to update the geometry (long-term problem) is described follows:

- Obtain maximum elastic stretch *λ*_*f*_ by solving the short time-scale problem over a cardiac cycle using Eq. (17)
- Initialize ${\theta }_{i}^{1}s$ from the latest geometry
- Use *λ*_*f*,*h*_ from baseline model to estimate (*λ*_*f*_ − *λ*_*f*,*h*_)
- For *j* = 1 to *n*_*growthSteps*_
  - Explicitly update growth parameters: ${\theta }_{i}^{j+1}={\theta }_{i}^{j}+{k}_{i}({\theta }_{i}^{j},\,{\lambda }_{f})({\lambda }_{f}-{\lambda }_{f,h})$
  - Update ***F***_*g*_ using $${\theta }_{i}^{j+1}$$
  - Solve for new geometry using Eq. (18).

Similar to our previous approach^8^, residual stresses induced by the incompatible growth tensor were removed after each growth cycle. As a consequence of this ***F*** = ***F***_*e*_ in Eq. (17a).

### Simulation cases

Two cases differing in terms of the prescribed activation initiation location were simulated. These cases are, namely,

- *Normal*: activation was initiated at the septum near the base,
- *Pacing*: activation was initiated at the LVFW near the base.

A schematic of the simulation timeline and pacing location is shown in Fig. 5. The homeostatic set point for the maximum elastic myofiber stretch *s*_*i*,*h*_ = *λ*_*f*,*h*_ in the growth constitutive model in Eq. (3) was prescribed using the local values obtained from the “*Normal*” case with septal activation. Deviations of the maximum elastic fiber stretch in the “*Pacing*” case from the homeostatic values were used as growth stimuli.

### Calibration of G&R simulations

**Figure 6:**
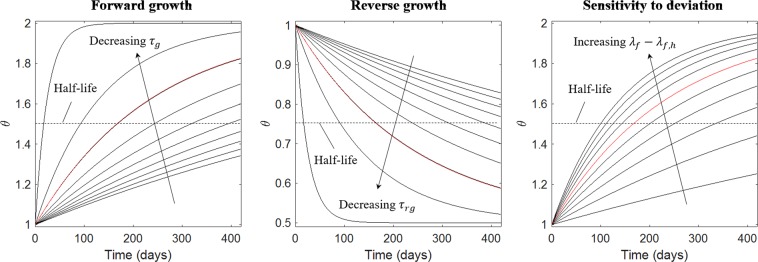
Effect of varying G&R parameters. Changes in Left: *τ*_*g*_ from 40.0 to 1.0 for *λ*_*f*_ − *λ*_*f*,*h*_ = 0.06; Middle: *τ*_*g*_ from 40.0 to 1.0 for *λ*_*f*_ − *λ*_*f*,*h*_ = −0.06; Right: *λ*_*f*_ − *λ*_*f*,*h*_ from 0.01 to 0.1 for *τ*_*g*_ = 9.6. Red: Parameter values in Table 2.

Growth parameters used in the simulations are shown in Table 2. All other model parameters are given in the Appendix. Based on Eqs (3) and (4), half-life is given by

19 $${t}_{\frac{1}{2},j}={\tau }_{j}\,\mathrm{ln}\,(2)\frac{{\theta }_{max}-{\theta }_{min}}{{\lambda }_{f}-{\lambda }_{f,h}},$$

with subscript *j* = *g*,*rg* and *γ*_*g*,*i*_ = *γ*_*rg*,*i*_ = 1.0. Using a deviation of *λ*_*f*_ − *λ*_*f*,*h*_ = ±0.06, *τ*_*g*_ and *τ*_*rg*_ were initialized with a half-life of 170 days for *θ*_*s*_ and *θ*_*n*_. Given the exponential decay, this will produce an increase in *θ*_*s*_ and *θ*_*n*_ by 53% in 6 months. The net change in thickness also depends on the material properties of the body prescribed during growth deformations^8^ and hence, the simulated changes in wall thickness were expected to be lesser. Forward growth rates for *θ*_*f*_ and reverse growth rates for *θ*_*s*_ and *θ*_*n*_ were adjusted together to match LV dilation and early activated region thinning, respectively. The sensitivity of growth rates to *λ*_*f*_ − *λ*_*f*,*h*_ = ±0.06 is shown in Fig. 6. In six months, a change in *λ*_*f*_ − *λ*_*f*,*h*_ from 0.06 to 0.08 would result in corresponding changes in *θ*_*s*_ and *θ*_*n*_ from 53% to 63%.

**Table 2:** Model parameters.

Growth parameters						
Direction	*τ*_*g*_	*τ*_*g*_	*γ*	*θ*_*min*_	*θ*_0_	*θ*_*max*_
	Days	Days	Days	(no units)	(no units)	(no units)
*θ*_*f*_	3.8	9.6	1.0	0.5	1.0	2.0
*θ*_*s*_	9.6	3.8	1.0	0.5	1.0	2.0
*θ*_*n*_	9.6	3.8	1.0	0.5	1.0	2.0

## Supplementary information

Appendix
