# Supplementary material for: Model of Anisotropic Reverse Cardiac Growth in Mechanical Dyssynchrony
Source: Sci Rep. 2019 Sep 3;9:12670. doi: 10.1038/s41598-019-48670-8 (PMC6722088; doi:10.1038/s41598-019-48670-8)

Model of Anisotropic Reverse Cardiac Growth in Mechanical Dyssynchrony

Jayavel Arumugam[1], Joy Mojumder[1], Ghassan Kassab[2], Lik Chuan Lee[1]

[1]Department of Mechanical Engineering, Michigan State University, East Lansing, USA

[2]California Medical Innovations Institute, San Diego, CA, USA

Appendix

Short-term cardiac mechanics model parameters			
**Parameter**	**Value**	**Units**	**Comments**
$\boldsymbol{C}$	0.132	kPa	Exponential stiffness scaling factor
$\boldsymbol{b_f}$	29.0	--	Fiber stiffness factor
$\boldsymbol{b_{fs}}$	13.3	--	Transverse fiber stiffness
$\boldsymbol{b_{xx}}$	26.6	--	Transverse fiber shear stiffness
$\boldsymbol{k_{spring1}}$	0.50	kPa	Spring constant
$\boldsymbol{k_{spring2}}$	0.050	kPa	Spring constant
$\boldsymbol{T_{max}}$	175.2	kPa	Contractility
$\boldsymbol{C_{a0}}$	4.35	μM	Peak intracellular calcium concentration
$\boldsymbol{(C_{a0})_{max}}$	4.35	μM	Maximum peak intracellular calcium concentration
$\boldsymbol{B}$	5.75	μM^{-1}	Controls peak isometric tension-sarcomere relation
$\boldsymbol{l_0}$	1.58	μM	Sarcomere length at which no tension develops
$\boldsymbol{l_{s0}}$	1.85	μM	Reference sarcomere length
$\boldsymbol{t_0}$	250	ms	Time to maximum active tension
$\boldsymbol{m}$	290	ms.μM^{-1}	Controls sarcomere length-dependent relaxation
$\boldsymbol{b}$	-275	ms	Controls sarcomere length-dependent relaxation

Long-term cardiac mechanics model parameters			
**Parameter**	**Value**	**Units**	**Comments**
$\boldsymbol{C}$	0.1	kPa	Exponential stiffness scaling factor
$\boldsymbol{b_f}$	29.0	--	Fiber stiffness factor
$\boldsymbol{b_{fs}}$	13.3	--	Transverse fiber stiffness factor
$\boldsymbol{b_{xx}}$	26.6	--	Transverse fiber shear stiffness factor
$\boldsymbol{k_{spring1}}$	0.100	kPa	Spring constant
$\boldsymbol{n_{growthSteps}}$	15	--	Number of growth steps across 2 months

Cardiac electrophysiology model parameters			
**Parameter**	**Value**	**Units**	**Comments**
d_{iso}	0.15	$cm^2.ms^{-1}$	Myocardial conduction
d_{ani}	0.7	$cm^2.ms^{-1}$	Conduction along myofiber
c	8	--	Controls action potential dynamics
α	0.01	--	Controls action potential dynamics
b	0.15	--	Controls action potential dynamics
γ	0.002	--	Controls cardiac restitution
μ_1	0.2	--	Controls cardiac restitution
μ_2	0.3	--	Controls cardiac restitution

Windkessel model parameters			
**Parameter**	**Value**	**Units**	**Comments**
P_{aor}	60	mmHg	Aortic valve opening pressure
P_{pul}	12	mmHg	Pulmonary valve opening pressure
P_{mv}	5	mmHg	Mitral valve opening pressure
P_{tr}	4	mmHg	Tricuspid valve opening pressure
C_{aor}	0.007	ml.Pa	Aortic compliance
C_{pul}	0.01	ml.Pa	Pulmonary compliance
R_{pul}	6e4	$Pa.ms.ml^{-1}$	Pulmonary peripheral resistance
R_{sys}	1e5	$Pa.ms.ml^{-1}$	Systemic peripheral resistance
R_{aor}	5.5e3	$Pa.ms.ml^{-1}$	Aortic resistance
R_{pa}	5e2	$Pa.ms.ml^{-1}$	Pulmonary artery resistance
P_{psys}	0.6	kPa	Peripheral systemic pressure
P_{ppul}	0.2	kPa	Pulmonary terminal pressure

Additional Simulation of LVFW pacing using same forward rates in reverse direction but different rates in fiber and cross-fiber directions:

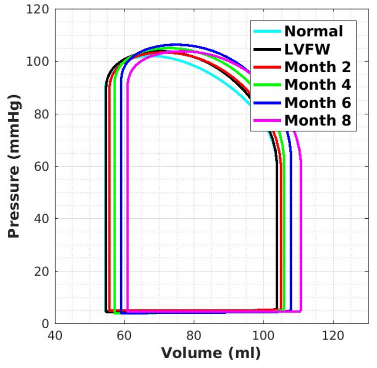

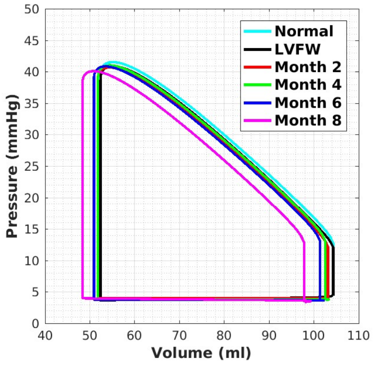

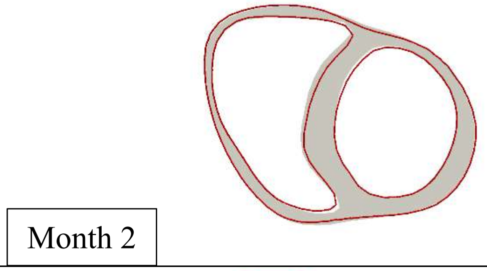

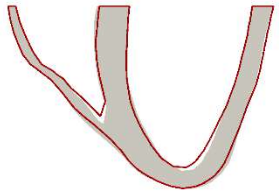

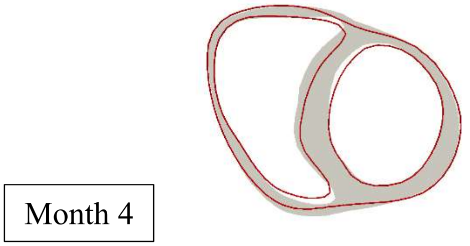

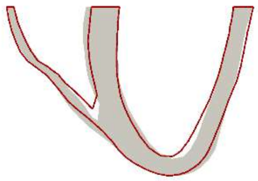

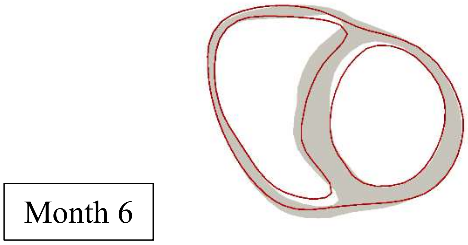

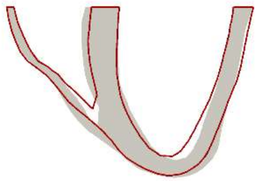

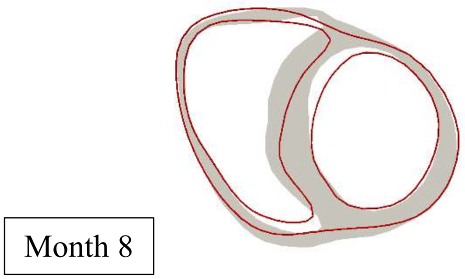

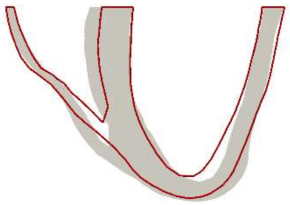

Additional Simulations of Pressure Overload

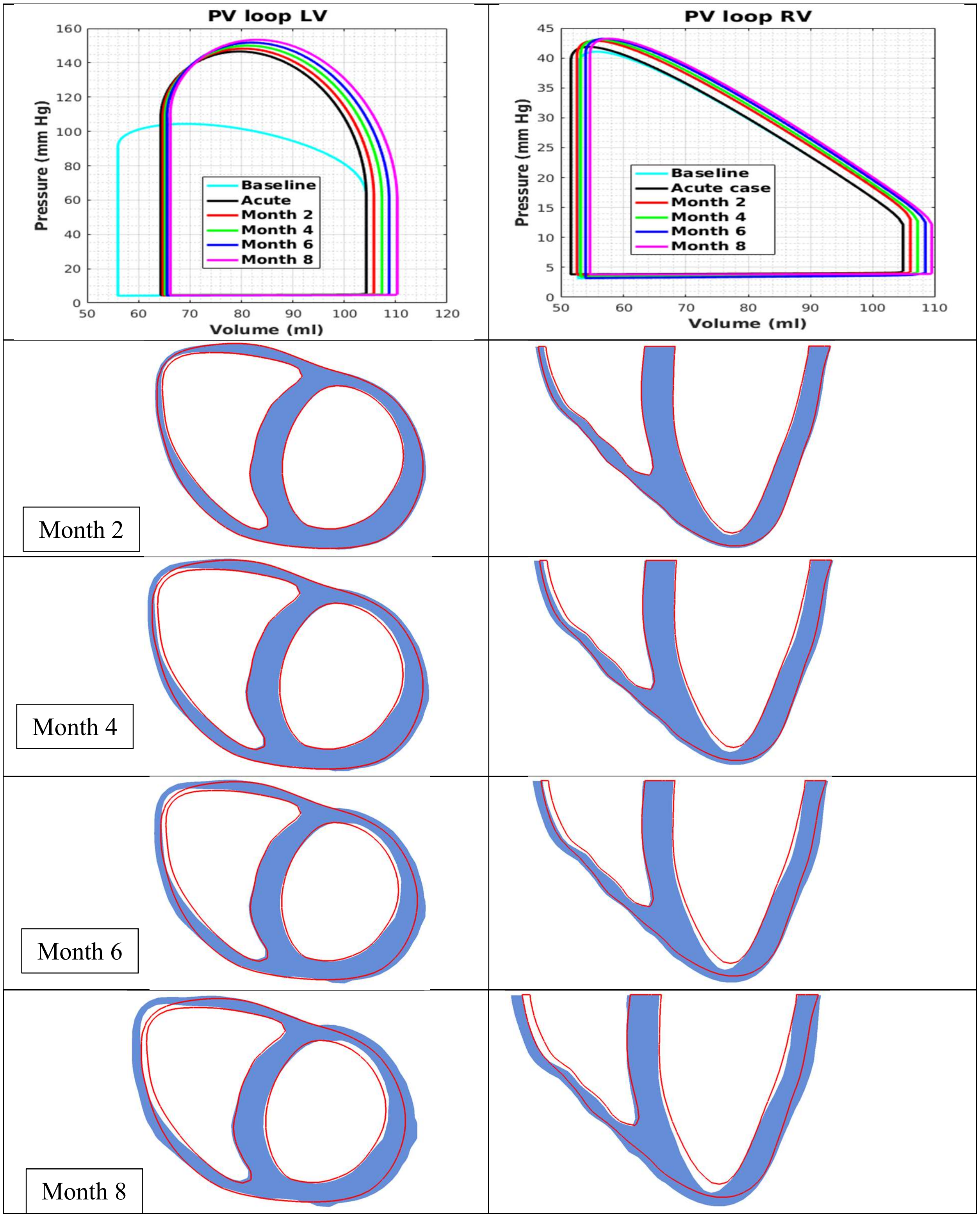

Additional Simulations of Volume Overload

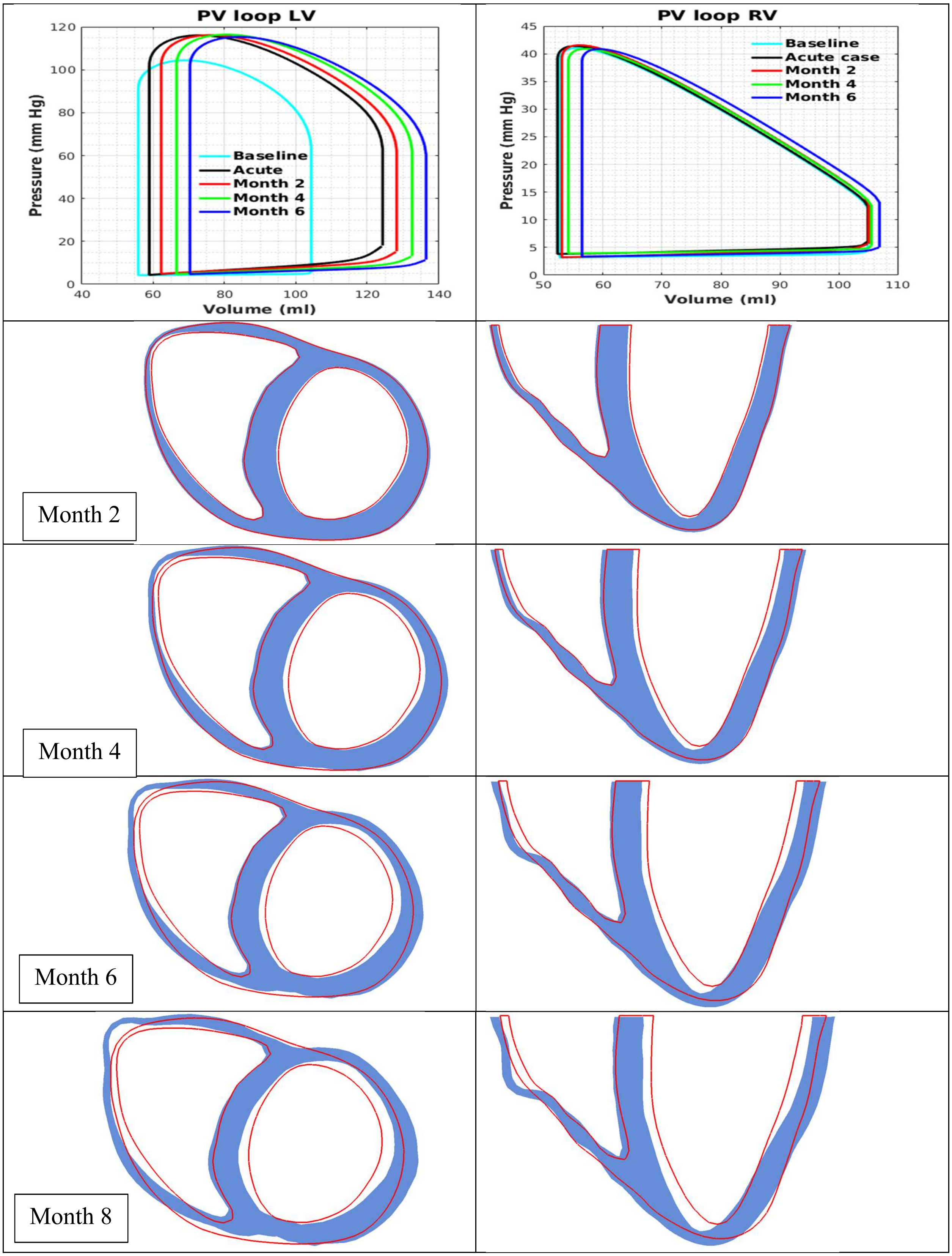

Supplement: Supplementary file 1 — Appendix [file 41598_2019_48670_MOESM1_ESM.pdf]
